# Changes in MicroRNA Expression during Rabbit Hemorrhagic Disease Virus (RHDV) Infection

**DOI:** 10.3390/v12090965

**Published:** 2020-08-31

**Authors:** Beata Hukowska-Szematowicz, Agata Maciejak-Jastrzębska, Małgorzata Blatkiewicz, Karolina Maciak, Monika Góra, Joanna Janiszewska, Beata Burzyńska

**Affiliations:** 1 Institute of Biology, University of Szczecin, 71-412 Szczecin, Poland; 2 Molecular Biology and Biotechnology Center, University of Szczecin, 71-412 Szczecin, Poland; 3 Department of Clinical Chemistry and Laboratory Diagnostics, Medical University of Warsaw, 02-091 Warsaw, Poland; amaciejak@wum.edu.pl; 4 Department of Medical Biology, Pomeranian Medical University, 70-204 Szczecin, Poland; blatkiewicz.malgorzata@gmail.com; 5 Institute of Biochemistry and Biophysics, Polish Academy of Sciences, 02-106 Warsaw, Poland; kmaciak@ibb.waw.pl (K.M.); mgora@ibb.waw.pl (M.G.); atka@ibb.waw.pl (B.B.); 6 Institute of Human Genetics, Polish Academy of Sciences, 60-479 Poznan, Poland; jjanisz@man.poznan.pl

**Keywords:** microRNA, rabbit hemorrhagic disease virus (RHDV), liver, STRING analysis

## Abstract

Current knowledge on the role of microRNAs (miRNAs) in rabbit hemorrhagic disease virus (RHDV) infection and the pathogenesis of rabbit hemorrhagic disease (RHD) is still limited. RHDV replicates in the liver, causing hepatic necrosis and liver failure. MiRNAs are a class of short RNA molecules, and their expression profiles vary over the course of diseases, both in the tissue environment and in the bloodstream. This paper evaluates the expression of miRNAs in the liver tissue (ocu-miR-122-5p, ocu-miR-155-5p, and ocu-miR-16b-5p) and serum (ocu-miR-122-5p) of rabbits experimentally infected with RHDV. The expression levels of ocu-miR-122-5p, ocu-miR-155-5p, and ocu-miR-16b-5p in liver tissue were determined using reverse transcription quantitative real-time PCR (RT-qPCR), and the expression level of circulating ocu-miR-122-5p was established using droplet digital PCR (ddPCR). The expression levels of ocu-miR-155-5p and ocu-miR-16b-5p were significantly higher in the infected rabbits compared to the healthy rabbits (a fold-change of 5.8 and 2.5, respectively). The expression of ocu-miR-122-5p was not significantly different in the liver tissue from the infected rabbits compared to the healthy rabbits (*p* = 0.990), while the absolute expression level of the circulating ocu-miR-122-5p was significantly higher in the infected rabbits than in the healthy rabbits (*p* < 0.0001). Furthermore, a functional analysis showed that ocu-miR-155-5p, ocu-miR-16b-5p, and ocu-miR-122-5p can regulate the expression of genes involved in processes correlated with acute liver failure (ALF) in rabbits. Search tool for the retrieval of interacting genes/proteins (STRING) analysis showed that the potential target genes of the three selected miRNAs may interact with each other in different pathways. The results indicate the roles of these miRNAs in RHDV infection and over the course of RHD and may reflect hepatic inflammation and impairment/dysfunction in RHD.

## 1. Introduction

Rabbit hemorrhagic disease virus (RHDV), genotype GI.1, belongs to the family *Caliciviridae*, genus *Lagovirus* [1], and is an etiological agent of rabbit hemorrhagic disease (RHD). It was first reported in China in 1984 in rabbits imported from Germany and spread over the entire country in less than a year, causing large-scale rabbit deaths [2]. The main driving force behind the genetic variability of this virus is RNA-dependent RNA polymerase (RdRp) [3]. This enzyme determines the virus’s fidelity, as well as the speed of its replication and mutation, and conditions its adaptation to the environment and new hosts. An analysis of the genetic variability of *Lagovirus europaeus* GI.1 (RHDV) and GI.2 (RHDV2) on the basis of the RdRp coding gene indicated growing genetic variability between the strains, both in time and location. Moreover, phylogenetic analysis showed a division of the 105 strains *Lagovirus europaeus* into seven groups, determined by their chronology, geographical location, and evolutionary events, such as mutation and recombinations [3]. Currently, this disease affects rabbit populations worldwide, and although more than 30 years have passed since the first outbreak of rabbit hemorrhagic disease, the mechanisms underlying the pathogenesis of the disease are not yet fully understood. RHD is a highly infectious and deadly disease, most frequently occurring in three clinical forms: Hyperacute, acute, and chronic. The death of infected rabbits occurs approximately 1–3 days after infection, and the mortality rate often reaches 100% [4]. The most intense changes over the course of RHD are found in the liver (the site of viral replication), with the first RHDV molecules in the liver detected as early as the first hours of infection. In addition, the number of infected hepatocytes increases over the course of the disease and is highest between 24 and 36 h post infection [4,5,6]. Infection results in an increased rate of liver cell death via necrosis [7] and apoptosis [5,8,9]. The pathogenesis of the disease also includes the apoptosis of granulocytes and peripheral blood lymphocytes in rabbits, which is more intense in lymphocytes [10,11,12,13]. RHD-related death can also be caused directly by systemic hemorrhagic diathesis and disseminated intravascular coagulation (DIC) [4], likely due to liver cell damage from apoptosis induced by RHDV [8,14]. A study on the normal rabbit kidney epithelial (RK13) and human liver hepatocellular (HepG2) cell lines [15] showed that RHDV non-structural protein 6 (NSP6) can induce apoptosis in host cells and is likely an important factor of RHD pathogenesis. In addition, it has been found that N-acetylcysteine [9], cardiotrophin [16], and melatonin [17] can alleviate liver damage and prolong the lives of rabbits infected with RHDV, likely due to the induction of various antiapoptotic agents. An important role in the pathogenesis of RHD is also played by innate and adaptive immunity [10,13,18], including peripheral blood leukocytes [19,20]. The expression of the genes encoding cytokines interleukin (IL): IL-6, IL-8, IL-10, tumor necrosis factor α (TNF-α), TNF-β, interferon-γ (IFN-γ), and granulocyte-macrophage colony-stimulating factor (GM-CSF) is intensified in the leukocytes in rabbit peripheral blood, with the level of expression depending on the stage of the disease and affecting the survival of infected rabbits [19,20]. A recent study by Semerjyan et al. [21] showed that RHDV induces pathology in leukocytes due to hyperactivation with a shift towards immature stages of different cell lines. Quantitative cellular changes were accompanied by an increase in the levels of inflammatory cytokines [21]. 

MicroRNAs (miRNAs) are a class of short (20–25 nucleotide) single-stranded RNA molecules, whose task is to regulate gene expression [22]. Abnormalities within cellular miRNA have a direct impact on cellular processes and, thus, form part of the pathogenesis of many human and animal diseases, such as those affecting the liver [23,24,25]. The miRNA expression profile varies over the course of different diseases, not only in the affected organ, e.g., the liver, but also in the serum or plasma of infected humans and animals [26,27,28,29,30]. MiRNA in serum or plasma is called circulating miRNA, which, despite being formed inside the cell, can be released into the bloodstream [31]. The mechanism for the release of miRNAs outside the cell is not yet fully understood, but three main theories of the origin of circulating miRNAs have been proposed [31]. The first is based on the view that the presence of miRNAs in blood is an undesirable product of cell death and occurs as a result of the energy-neutral “leakage” of cellular miRNAs. The second hypothesis is that miRNAs are actively released from the cell by microvesicles and exosomes. The third theory assumes that the active and selective release of miRNAs in an independent and microvesicle-free form as a result of a cellular responses to various stimuli [31].

Liver specific miR-122-5p [32,33,34], miR-155-5p, involved in inflammatory and immunological processes [34,35,36,37], and miR-16-5p, which is involved in apoptosis, immunological processes, differentiation, and the regulation of cell growth [38,39], were selected for the study. These three miRNAs have proven significance in liver diseases, particularly in acute liver failure (ALF). The present results evaluate the expression of miRNAs in the liver tissue (ocu-miR-122-5p, ocu-miR-155-5p, and ocu-miR-16b-5p) and serum (ocu-miR-122-5p) of rabbits experimentally infected with RHDV.

## 2. Materials and Methods

### 2.1. Animal and Rabbit Hemorrhagic Disease Virus (RHDV)

This study was conducted on 20 healthy conventional rabbits (CV) (a mixed-breed with both sexes), weighing 2.80–3.00 kg, aged 12 weeks. The rabbits were purchased from a licensed farm operating under the constant supervision of veterinary and animal husbandry staff. The animals were acclimatized to the vivarium for at least 5 days after being transported and were randomly assigned into 2 groups: Rabbits infected with RHDV (referred to as the infected rabbits) and control rabbits (referred to as the healthy rabbits). The rabbits were fed with certified rabbit food (Motycz, Poland) and water ad libitum. Before the experiment, all rabbits were tested for anti-RHD antibodies using enzyme-linked immunosorbent assay (ELISA). During the experiment, the zoohygienic parameters were checked every 24 h at 8.00 am. The rabbits of the infected group (*n* = 10) were intramuscularly given (hind limb muscles) RHDV strain Erfurt GI.1a (RHDVa). The control group healthy rabbits (*n* = 10) were given phosphate buffered saline (PBS) as a placebo in the same manner. The Erfurt RHDV (German strain) has been described as an antigenic variant of RHDV (G6/RHDVa). According to the new classification system, it is identified as *Lagovirus europaeus* GI.1a [1]. The virus was isolated from an animal that had died and was found in the field in the country of origin. The virus was identified using RT-qPCR [10]. A liver homogenate was prepared in the laboratory, and the rabbits were infected to multiply the virus. After their deaths, their livers were prepared as a 20% homogenate and centrifuged at 1600 G, followed by treatment with 10% chloroform for 60 min and another centrifuging step. The final suspension was prepared in PBS at 1:1 ratio. All prepared antigens contained the same number of viral particles at 1.310 g/cm3 to 1.340 g/cm3 [40]. The experiment was approved by the local ethics committee in Szczecin (No. 1/2009, 26 January 2009). During the experiment, the clinical conditions of the infected and control animals were assessed. Possible clinical symptoms in the infected animals and mortality were recorded.

### 2.2. Tissue Samples

Liver tissues were sampled post mortem and clinically defined. For healthy rabbits, the liver tissue was taken after euthanization. Each liver was cleared of blood by perfusion with a cold water–salt solution, immediately placed in an RNAlater stabilization reagent (Qiagen, Hilden, Germany) according to the manufacturer’s protocol, and stored at −80 °C until RNA extraction. 

### 2.3. Serum Samples

Blood (1 mL) was collected through a venflon using a tube inserted into an ear vein without an anticoagulant. From each infected rabbit, blood was collected before virus administration at 0 h after virus administration at 8 h, 12 h, 24 h, 36 h, 48 h, 52 h, 56 h, 60 h, and 72 h. Blood from healthy rabbits (*n* = 10) was taken only on the first day at 0 h. Blood samples were left at room temperature for 30 min to allow complete coagulation. Then, the coagulated samples were cooled to 4 °C and centrifuged at 1500 G for 15 min to separate the serum. The serum was transferred to a new cryotube with care to not disturb the buffy coat and immediately frozen at −80 °C until RNA extraction.

### 2.4. RNA Isolation from Tissue Samples

The total RNA, including miRNA, was extracted from 50 mg of liver tissue using an miRNeasy Mini Kit (Qiagen, Germany) according to the manufacturer’s instructions. Potential genomic DNA contamination was removed by an RNase-Free DNase Set (Qiagen, Germany). RNA concentration and quality were determined using a NanoDrop 2000 spectrophotometer (Thermo Fisher Scientific, Waltham, MA, USA).

### 2.5. RNA Isolation from Serum Samples

Before RNA extraction, all serum samples were thawed completely on ice followed by centrifugation once at 20.000 G for 15 min at 4 °C to remove the remaining cell debris. Total RNA, including miRNA, was isolated from 200 µL of serum aliquots using an miRNeasy Serum/Plasma Kit (Qiagen, Germany) according to the manufacturer’s protocol. 

### 2.6. miRNA Polyadenylation and Reverse Transcription (RT) Reaction

Isolated total RNA was used as a template for the synthesis of cDNA via a miRCURY LNATM Universal RT miRNA PCR Starter Kit (Exiqon A/S, Vedbæk, Denmark) with the addition of spike in UniSp6 (Exiqon A/S, Denmark). The RT reaction was performed according to the manufacturer’s instructions. In the tissue samples, 5 ng/μL of total RNA was used for cDNA synthesis. In the serum samples, the input RNA for RT reactions was based on the starting material volume. The cycling conditions for the RT reaction were as follows: Incubation for 60 min at 42 °C, heat-inactivation of the reverse transcriptase for 5 min at 95 °C, and immediate cooling to 4 °C. Then, cDNA was stored at −20 °C until further experiments. 

### 2.7. Quantification of ocu-miR-122-5p, ocu-miR-155-5p, and ocu-miR-16b-5p in Liver Samples Using Quantitative Real-Time PCR (qPCR) and Data Analysis

The expression of ocu-miR-122-5p, ocu-miR-155-5p, and ocu-miR-16b-5p was determined by the qPCR reactions in tissue samples using miRNA LNA™ PCR primer sets (Exiqon A/S, Denmark) and the ExiLENT SYBR^®^ Green master mix (Exiqon A/S, Denmark) from the starter kit, according to the manufacturer’s instructions. cDNA templates were diluted 80-fold in RNase-free water. The nucleotide sequences of the analyzed miRNAs are presented in Table 1. The amplification of the selected miRNA was performed using a LightCycler^®^480 Real-Time PCR system (Roche, Basel, Switzerland) with the following cycling conditions: Polymerase activation/denaturation 95 °C for 10 min and 45 amplification cycles at 95 °C for 10 s and 60 °C for 1 min, with a ramp-rate of 1.6 °C/s. To verify the non-specific products, a melting curve analysis was carried out at the end of the amplification cycle. The qPCR data were normalized using ocu-let-7a-5p and ocu-miR-103a-3p as stable reference genes. Candidate reference genes were selected on the basis of a previous study [41] and an evaluation of their stability under experimental conditions using the geNorm, NormFinder, and BestKeeper algorithms [42].

### 2.8. Quantity of miRNAs in Serum Samples Measured by ddPCR and Data Analysis

The quantity of ocu-miR-122-5p in the serum samples was measured by the droplet digital PCR (ddPCR) method using a QX200™ Droplet Digital™ PCR (ddPCR™) System (Bio-Rad, Philadelphia, PA, USA). Despite this attempt, the reaction conditions for ocu-miR-155-5p and ocu-miR-16b-5p determination in the serum samples could not be optimized. Their expression level in the serum was poorly detectable. For this reason, the studies in this article only present the results for ocu-miR-122-5p, for which satisfactory optimization and expression results were obtained. The cDNA templates were diluted 20-fold, and a 22 µL PCR reaction mixture was added according to the manufacturer’s protocol for miRNA PCR profiling using miRNA LNA™ PCR primer sets (Exiqon A/S, Denmark) and EvaGreen (Bio-Rad, USA) with a QX200™ Droplet Digital™ PCR System (Bio-Rad, USA). The PCR amplification conditions were 95 °C for 5 min, 40 amplification cycles at 95 °C for 30 s, and 56 °C for 1 min, with a ramp-rate of 1.6 °C/s and final dye stabilization at 4 °C for 5 min and 90 °C for 5 min. After the PCR reaction, the droplets were read by a QX200™ Droplet Reader (Bio-Rad, USA). The results were then analyzed using QuantaSoft™ v.1.7.4.0917 and QuantaSoft™ Analysis Pro v.1.0.596 (Bio-Rad, USA). A no template control (NTC) was included for the ddPCR reaction mix. The miRNA concentrations were calculated using Poisson statistics and background-corrected based on the signals observed in the NTC. The absolute miRNA levels were corrected for the input amount of serum and presented as the copies/µL serum.

### 2.9. In Silico Prediction of miR-122-5p, miR-155-5p, and miR-16b-5p Target Genes in Oryctolagus cuniculus

The MiRTarBase database was used to select target genes previously validated by RT-qPCR, Western blot, or a reporter assay in other species [43]. Next, the set of genes was used to conduct a gene onthology (GO) analysis via a GO Enrichment Analysis powered by protein annotation through evolutionary relationship (PANTHER) [44,45]. The experiment was as follows: Analysis type: PANTHER overrepresentation test; reference list: All homo sapiens genes in the database; annotation data set: GO biological process complete; test type: Fisher’s exact; and correction: Calculate false discovery rate (FDR). From all the processes with FDR *p* < 0.05, those that were correlated with liver diseases in humans and animals were used for further steps. The 3′UTR sequences of the *Oryctolagus cuniculus* genes involved in the selected processes were assessed to determine if they featured binding sites for ocu-miR-122-5p, ocu-miR-155-5p, or ocu-miR-16b-5p using the TargetScan database [46]. The last step was the visualization of protein interactions based on the search tool for the retrieval of interacting genes/proteins STRING database for *Oryctolagus cuniculus* [47]. The analysis settings were as follows: Meaning of network edges; action mode; active interaction sources: Textmining, experiments, databases, co-expression, neighborhood, gene fusion, and co-occurrence; minimum required interaction score: Medium confidence; and max number of interactors to show: None.

### 2.10. Statistical Analysis

In liver tissues, the relative expression of ocu-miR-122-5p, ocu-miR-155-5p, and ocu-miR-16b-5p between the infected and healthy rabbits, normalized to ocu-let-7a-5p and ocu-miR-103a-3p, was calculated using the Pfaffl model [48] and the Relative Expression Software Tool 384, version 2 (REST-384) [49]. In the serum samples, the data obtained from the ddPCR experiments were analyzed using Statistica 13.0 PL (StatSoft, Krakow, Poland). Prior to the comparative analyses, a Kolmogorov–Smirnov test (K-S) was carried out to determine whether the experimental data were subject to normal distribution. Then, the data were log transformed. To determine if there are differences in the post-infection time in RHDV infected rabbits, a Kaplan–Meier analysis (KM) was performed. Finally, a Mann–Whitney test (MW) was performed to detect whether there were differences in the absolute expression level of circulating ocu-miR-122-5p between the healthy and infected rabbits. *p* < 0.05 was considered statistically significant. 

## 3. Results

### 3.1. Clinical Signs of RHD

During the experiment, only a few animals showed clinical signs of RHD (apathy and gelled feces), while the remaining rabbits died suddenly. The mortality rate was 100%. The survival time of the animals varied. Thus, using the Kaplan–Meier method, infected rabbits were divided into two groups based on their post-infection survival time. Infected rabbits were divided into groups to assess if there was a relationship between the expression level of ocu-miR-122-5p in the serum and the survival time of the animals. The first group (infected one (I1), with a shorter survival time) consisted of rabbits that died up to the 56th hour of the experiment (five died between 24/36 h and two at 56 h). The second group (infected two (I2), with a longer survival time) included rabbits that died after 60 h (72 h) (three rabbits). The division of the rabbits into groups I1 and I2 was used only to analyze the expression of ocu-miR-122-5p in serum at the designated time points, whereas the liver tissue was only analyzed post mortem.

### 3.2. Tissue Level of ocu-miR-122-5p, ocu-miR-155-5p, and ocu-miR-16b-5p during RHDV Infection

**Figure 1:**
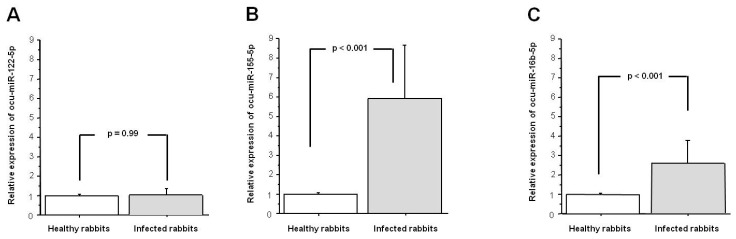
Expression of (**A**) ocu-miR-122-5p; (**B**) ocu-miR-155-5p; and (**C**) ocu-miR-16b-5p in the liver tissue from infected rabbit hemorrhagic disease virus (RHDV) rabbits in comparison with the healthy rabbits. Relative expression data were normalized to use ocu-let-7a-5p and ocu-miR-103a-3p as stable reference genes. Results are shown as the mean ± standard error, and *p* < 0.05 was considered significant.

We examined the relative expression levels of ocu-miR-122-5p, ocu-miR-155-5p, and ocu-miR-16b-5p in the livers of the RHDV infected rabbits and compared these findings with the levels obtained in the tissues of the healthy rabbits. The relative expression of ocu-miR-122-5p was not significantly different in the liver tissue from the infected rabbits compared to the healthy rabbits (*p* = 0.990; Figure 1A). The expression level of ocu-miR-155-5p was significantly higher in the infected rabbits than in the healthy rabbits (a fold-change of 5.8, *p* < 0.001; Figure 1B). The relative expression of ocu-miR-16b-5p was also significantly higher in liver tissue from the infected rabbits compared with the healthy rabbits (a fold-change of 2.5, *p* < 0.001; Figure 1C).

### 3.3. Serum Level of ocu-miR-122-5p in Infected Rabbits Versus Healthy Rabbits

**Figure 2:**
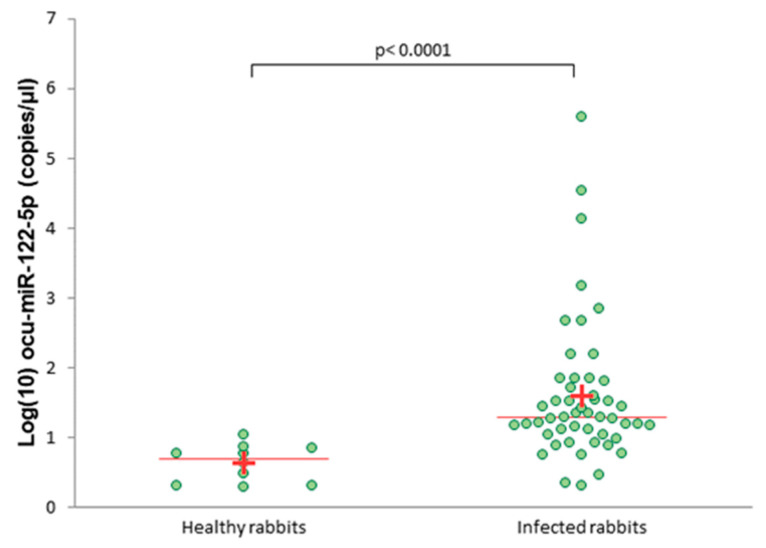
Absolute levels of serum ocu-miR-122-5p in healthy and infected rabbits measured by droplet digital PCR (ddPCR). A plus (+) indicates the average; a line indicates the median.

The absolute expression level of circulating ocu-miR-122-5p was significantly higher in the infected rabbits than in the healthy rabbits (*p* < 0.0001; Figure 2). 

**Figure 3:**
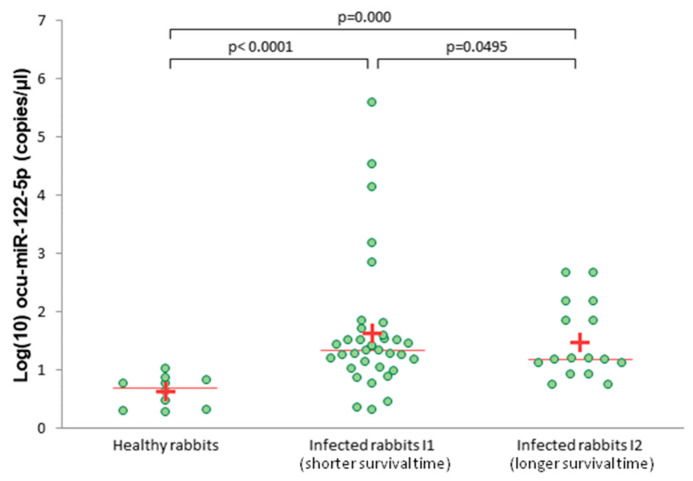
Absolute levels of serum ocu-miR-122-5p in healthy and infected rabbits (group I1 and I2), measured by ddPCR. A plus (+) indicates the average; a line indicates the median.

The expression level of ocu-miR-122-5p in the serum was significantly higher in both infected groups—I1 (shorter survival time) and I2 (longer survival time)—compared to the healthy rabbits (*p* < 0.0001 and *p* = 0.0001, respectively; Figure 3). In addition, the expression level of ocu-miR-122-5p in serum was significantly different between the I1 and I2 groups (*p* = 0.0495; Figure 3).

### 3.4. Prediction of Target Genes for the Three Selected MiRNAs in Oryctolagus cuniculus

**Figure 4:**
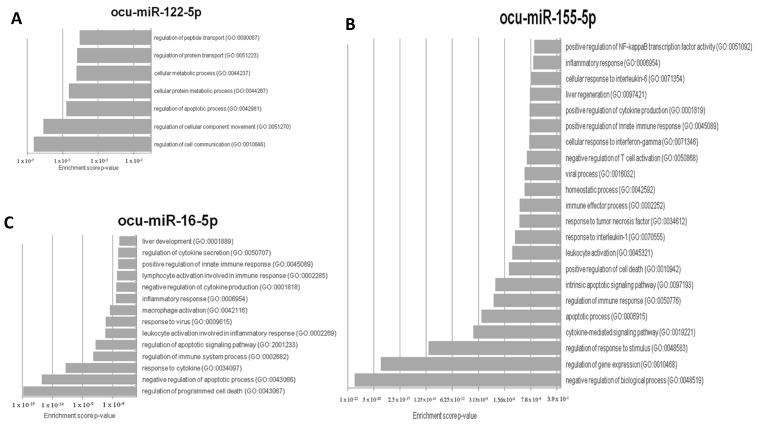
The most relevant rabbit hemorrhagic disease (RHD) pathogenesis and acute liver failure (ALF) gene onthology (GO) processes that can be regulated by (**A**) ocu-miR-122-5p, (**B**) ocu-miR-155-5p, and (**C**) ocu-miR-16b-5p.

To verify the importance of miR-122-5p, miR-16b-5p, and miR-155-5p in RHDV, an in silico analysis of putative target genes was conducted. Due to the inability to use one database to demonstrate the miRNA–mRNA interactions in *Oryctolagus cuniculus,* the following approach was selected. Firstly, mature sequences of these three miRNAs in *Oryctolagus cuniculus* and *Homo sapiens* were compared, and no differences were found. Therefore, we decided to use miRTarBase, which lists genes with validated miRNA–mRNA interactions by RT-qPCR or luciferase assays in *Homo sapiens*. Ultimately, three lists containing 51 target genes for miR-122-5p, 97 for miR-16b-5p, and 218 for miR-155-5p were created. Secondly, there was an attempt to determine the processes related to RHD that might be regulated by miR-122-5p, miR-16b-5p, and miR-155-5p. For this purpose, a GO analysis was conducted on the putative target genes for every miRNA separately. Thus, 80 processes for miR-122-5p, 1152 processes for miR-16b-5p, and 772 processes for miR-155-5p were identified. From these three groups, processes that correlated with RHD pathogenesis and ALF were chosen for further analysis: 7 for miR-122-5p, 22 for miR-155-5p, and 14 for miR-16b-5p (Figure 4A–C). Up to this point, all analyses were performed based on miRNA–mRNA interactions in *Homo sapiens*. To confirm if these regulations might also occur in *Oryctolagus cuniculus,* the TargetScan database was used. This tool enabled us to verify if the predicted binding sites were conserved in *Oryctolagus cuniculus.* Genes engaged in processes related to RHD and ALF were selected. Each miRNA–3′UTR interaction was checked independently. The TargetScan analysis revealed that 11 out of 38 genes for ocu-miR-122-5p, 63 out of 159 genes for ocu-miR-155-5p, and 30 out of 46 genes for ocu-miR-16b-5p have binding sites in 3′UTR in *Oryctolagus cuniculus* genes. The last step was the in silico validation of whether all 104 genes engage in the process presented in Figure 4 and are potentially regulated by ocu-miR-122-5p, ocu-miR-155-5p, and ocu-miR-16b-5p, as characterized by protein–protein interactions. For this analysis, the STRING database was used. STRING also allowed us to perform this analysis on *Oryctolagus cuniculus* proteins. 

**Figure 5:**
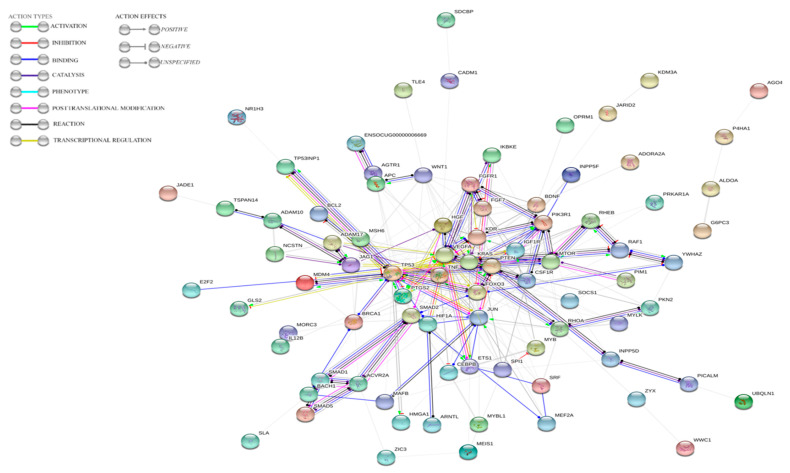
STRING analysis results indicating the dependences between genes potentially regulated by ocu-miR-122-5p, ocu-miR-155-5p, and ocu-miR-16b-5p.

The results indicated that almost all proteins potentially regulated by ocu-miR-122-5p, ocu-miR-155-5p, or ocu-miR-16b-5p with putative roles in processes connected to RHD and ALF are connected via common pathways (PPI enrichment *p*-value: *p* < 1.0 × 10^−16^) (Figure 5).

## 4. Discussion

RHDV is an etiological agent of RHD that affects rabbit populations worldwide. RHD is extremely lethal and highly contagious in both domestic and wild rabbits, with an acute and rapid course. The pathomechanism of RHDV infection is still not fully understood. Most RHDV-induced changes occur in the liver, the site of viral replication. Since a growing body of evidence indicates that miRNAs profoundly influence the course and pathogenesis of viral diseases, we hypothesized that the selected miRNA—ocu-miR-122-5p, ocu-miR-155-5p, and ocu-miR-16b-5p—may play a significant role in the response to RHDV infection as elements of RHD pathogenesis and may have potential use in the diagnosis of hepatic inflammation and impairment/dysfunction.

MiR-122 is a liver-specific molecule [32,33] that represents more than 70% of the miRNA in the livers of humans and animals, i.e., approximately 130.000 copies per cell, making this miRNA one of the most susceptible to expression in any tissue [32]. This molecule plays a key role in liver physiology, where it participates in hepatocyte differentiation, supports spontaneous regeneration, and takes part in liver homeostasis, lipid metabolism, and cholesterol synthesis [33]. In addition, many studies have shown the sensitivity and specificity of miR-122 as a circulating biomarker of liver injury in animals, such as chimpanzees [50], mice [30], rats [51,52], zebrafish [53], and dogs [54]. The literature also reported the potential use of miR-122 in humans as a biomarker for liver diseases caused, among others, by drug-induced liver injury (DILI) [29,55], hepatitis B and C [25,56], and ethanol consumption [57].

This study is the first attempt to determine the expression of ocu-miR-122-5p in the liver tissue and serum of rabbits infected with RHDV. There were no differences in the expression of ocu-miR-122-5p in the liver tissue of infected rabbits compared to healthy rabbits. On the one hand, this effect can be explained by the extremely fast course of the disease and the impossibility of spontaneous liver cell regeneration, as is the case with ALF [34]. The rate of viral replication in the liver also causes a loss of liver cell function and a breakdown of liver tissue integrity, leading to damage to this organ, which in turn leads to a release of ocu-miR-122-5p into the bloodstream [34,58]. Since miR-122-5p is released into the bloodstream due to various forms of liver damage and disease, the absolute level of this miRNA in the serum of rabbits infected with RHDV was determined in comparison with healthy rabbits using ddPCR. This method is a much more sensitive alternative to classical RT-qPCT and is, therefore, more suitable for circulating miRNA. In addition, it does not require a reference gene [59]. The absolute expression level of ocu-miR-122-5p in the serum in infected rabbits was significantly higher compared to that in healthy rabbits. This indicates the presence of liver injury and release of ocu-miR-122-5p into the bloodstream already at 12/24 h after infection and indicates a fast course of the disease. Moreover, an essential result of this study is the detection of significant differences in the ocu-miR-122-5p expression levels in serum between groups I1 (shorter survival time) and I2 (longer survival time). Possibly, the expression level of ocu-miR-122-5p in serum is associated with the survival time of the animals. In comparison to the group with shorter survival, the group with longer survival showed decreased expression levels of ocu-miR-122-5p in the infected rabbit serum. The inhibition of ocu-miR-122-5p expression in the serum may increase the time of survival for rabbits following RHDV infection. It can thus be hypothesized that the suppressed expression of ocu-miR-122-5p in rabbit serum could be due to the increased expression of hepatocyte growth factor (*HGF*) and its c-met receptor, as well as increased activity of the transcription factors signal transducer and activator of transcription 1 and 3 (*STAT1* and *STAT3*), which likely affect the liver’s regenerative capacity [14]. Previously researchers have indicated that the expression of miR-122 in the liver and serum is mutable and disease-dependent. In mice with acetaminophen (APAP) induced ALF, the expression of miR-122 was reduced in liver tissue but significantly increased in serum in a dose-dependent manner [30]. In chimpanzees after experimental hepatitis C virus (HCV) infection [50], a two–four-fold increase in miR-122 expression in liver and serum was observed at the beginning of infection (first 4 weeks); between 10 and 14 weeks after infection, the liver miR-122 levels decreased. In past studies on rats [51,52], the miR-122 levels in healthy liver tissue increased, while toxic substances and diseases increase miR-122 expression in the serum. In zebrafish [53], the toxic liver damage induced by tamoxifen or APAP increased the expression of hepatic specific miR-122 in liver tissue. In dogs with liver disease, the expression of miR-122 in serum was significantly higher than that in healthy dogs [54]. The expression of miR-122 was higher in the serum of humans with APAP-induced hepatotoxicity than that of the healthy controls, which demonstrated miR-122′s usefulness in diagnosing APAP-induced toxicity [29,55]. Studies evaluating the expression of miR-122 in serum for hepatitis B virus (HBV) and HCV infection showed that the expression of miR-122 in serum was higher compared to the healthy controls [25,56]. Studies analyzing miR-122 levels in the serum of healthy men before and after ethanol ingestion also showed an increase in the expression of this miR in serum [57].

The aforementioned reports, as well as our results, indicate that ocu-miR-122-5p in serum may potentially serve as a biomarker of liver damage from RHD. Moreover, the results of the GO analysis for ocu-miR-122-5p indicate that its potential role in the response to RHDV infection includes regulation of the expression of genes involved in the processes of hepatic homeostasis (which become disrupted in RHD) and, to a lesser extent, apoptosis (Figure 4A).

Of the three miRNAs analyzed in this study, ocu-miR-155-5p was the most abundantly expressed in rabbit liver tissue. The expression of miR-155 has been described in liver tissue, the thymus, spleen and immune system cell T and B lymphocytes, dendritic cells, and macrophages, regulating their functions and activation [35]. In the context of liver disease, miR-155 is expressed in liver cells, endothelial cells, and inflammatory cells, such as monocytes, NK cells, and macrophages [35,60,61]. The role of miR-155 in liver diseases is not yet fully understood and likely depends on the context of the disease [37,60]. It has been shown to be involved in the progression of liver inflammation [36]. In mice and humans, the expression of miR-155 in the liver is regulated by inflammatory cytokines such as TNF-a, IFN-b, and TNF-α [36]. A recent study on mice showed that the increased expression of miR-155 contributed to the pathogenesis of ALF via apoptosis by regulating the proinflammatory cytokine TNF [37]. The high expression of ocu-miR-155-5p in the liver tissue obtained in this study can be explained by the inflammatory responses in this organ caused by RHDV at 12/24 h post infection. Our findings are consistent with those of the STRING analysis and previous findings in the literature [14,17,60,62]. The inflammatory response induced a cascade of mediators such as TNF-α, TGF-β, IL-1β, IL-6, and IFN, as well as Toll-like receptor ligands (TLR) in monocytes/macrophages, which, in turn, induced an increase in miR-155 expression in the liver tissue (Figure 4B) [17,62]. Moreover, the high expression of this miRNA in liver tissue can be explained by its non-hepatocytic origin. Specifically, miR-155 may come from liver macrophage Browicz–Kupffer cells and immune system cells such as neutrophils and T and B lymphocytes (Figure 4B), which infiltrate the liver in RHD [14,61]. Studies on mice showed that alcohol consumption caused an increase in miR-155 expression in Browicz–Kupffer cells, which, in turn, led to an increase in TNF-α production by these cells. Other studies on mice demonstrated the protective role of miR-155 in liver tissue [60]. After inducing liver damage in mice via concanavaline (CoA), previous authors observed a 20–fold increase in miR-155 expression [60]. In the same study, the patients with hepatitis and cirrhosis showed a significant increase in miR-155 expression in the liver but decreased expression of this miRNA in peripheral blood mononuclear cells (PMBCs). The authors [60] suggested that appropriate expression of miR-155 in the liver and inflammatory cells may be crucial for an effective inflammatory response in a damaged liver, which can also hypothetically occur over the course of RHD. Interestingly, apoptotic genes can also be involved in the regulation of livers damaged during RHDV infection (Figure 4B). 

The biological role of miR-16-5p is to regulate apoptosis, the immunological process, proliferation, and cell cycle modulation [38,39]. MiR-16 suppresses the secretion and expression of the mRNAs of proinflammatory factors such as IL-6 and TNF-α, while increasing the secretion and expression of mRNA IL-10 [39]. Over the course of RHD infection, it has been proven that liver cell death occurs through apoptosis (among other causes). Between 12 h and 36 h, the expression of TNF-α and the ligand for the FAS receptor (FasL) increased. At the same time, this indicates the activation of apoptosis through the receptor pathway (external) [14], which could hypothetically be accompanied by an increase in ocu-miR-16b-5p expression, thereby modulating the apoptosis of liver cells (Figure 4C). On the other hand, the inhibition of miR-16 in liver cells caused a decrease in apoptosis and the production of TNF, which demonstrates the proapoptotic role of miR-16 [38]. Moreover, it has been shown that rabbit hepatocytes at 36-48 h post infection indicate an increased level of caspase-3 activity and significantly higher expression of the proapoptotic Bax protein compared to the Bcl-2 antiapoptotic protein [9,14,17]. 

We suggest that in the pathogenesis of RHD, the ocu-miRNA-16b-5p molecule is essential for multiple processes. On the one hand, this miRNA promotes liver cell apoptosis in response to viral infection. On the other hand, the expression of ocu-miRNA-16b-5p is crucial for an effective inflammatory response in damaged liver tissue (Figure 4C). Moreover, ocu-miRNA-16b-5p is required for cell proliferation during the liver regeneration process, especially in the parenchyma as well as in the restoration of protein-forming and metabolic functions [63].

Our research suggests that ocu-miR-155-5p and ocu-miR-16b-5p, whose expression is enhanced in rabbit liver tissue, can regulate inflammation and apoptosis processes, which was established by a functional analysis. We assume that the potential role of ocu-miR-122-5p (whose increased expression we observed only in rabbit serum) in response to RHDV infection is associated with regulation of the target genes involved in the processes of hepatic homeostasis, which are disrupted in RHD. The inhibition of ocu-miR-122-5p expression in serum possibly increases the time of survival and may be potentially used as a biomarker of liver damage in rabbits following RHDV infection. All three miRNAs have the same mature sequences in *Homo sapiens* and *Oryctolagus cuniculus*. Therefore, the target genes regulated by the analyzed microRNAs may play similar roles in hepatic diseases in both humans and rabbits. This work contributes to a better understanding of the relevant pathomechanisms, which is necessary to control the spread of RHDV in rabbits. Furthermore, the data in this study may be helpful in optimizing the therapeutic approaches for patients with ALF. Further data collection using a larger group of animals is required to determine precisely how miRNAs and their target genes modulate RHDV infection.

## 5. Conclusions

This work shows, for the first time, changes in the expression of ocu-miR-122-5p, ocu-miR-155-5p, and ocu-miR-16b-5p during RHDV infection. Our data suggest that alterations in ocu-miR-155-5p and ocu-miR-16b-5p expression in liver tissue and ocu-miR-122-5p in serum reflect changes in the inflammatory response of the liver and its injury over the course of RHD pathology. The abundant expression of ocu-miR-155-5p and ocu-miR-16-5p in liver tissue and ocu-miR-122-5p in serum is likely the result of liver damage in response to RHDV infection. The functional analysis showed that the analyzed miRNAs (ocu-miR-122-5p, ocu-miR-155-5p, and ocu-miR-16b-5p) are involved in biological processes such as inflammatory responses, apoptotic processes, and viral responses, which are crucial for ALF during RHDV infection. The binding sites for the analyzed miRNA were found in the 3′UTR sequences of the genes engaged in these processes in *Oryctolagus cuniculus* and might indicate the role of ocu-miR-155-5p and ocu-miR-16b-5p in responses during RHDV infection. Combining these possibilities, further research is necessary to determine the role of miRNA, as considerable elements of the pathogenesis of RHD and potential biomarkers of inflammation and liver injury/dysfunction.

## Figures and Tables

**Table 1:** Sequences of microRNAs (miRNAs).

miRNAs	Sequences
miRNAs tested	
ocu-miR-122-5p	5′UGGAGUGUGACAAUGGUGUUUG3′
ocu-miR-155-5p	5′UUAAUGCUAAUCGUGAUAGGGGUU3′
ocu-miR-16b-5p	5′UAGCAGCACGUAAAUAUUGGCGU3′
reference miRNAs	
ocu-let-7a-5p	5′UGAGGUAGUAGGUUGUAUAGUU3′
ocu-miR-103a-3p	5′AGCAGCAUUGUACAGGGCUAUGA3′

ocu: *Oryctolagus cuniculus*; miR: MicroRNA.
